# Digital health interventions targeting psychological health in parents of children with autism spectrum disorder: a scoping review

**DOI:** 10.1186/s40359-025-03219-5

**Published:** 2025-10-10

**Authors:** Binbin Ji, Intan Maharani Sulistyawati Batubara, Janene Batten, Xinyi Peng, Sanmei Chen, Zhao Ni

**Affiliations:** 1 School of Nursing, Hunan University of Chinese Medicine, Changsha, Hunan Province China; 2 School of Nursing, Yale University, Orange, CT United States; 3 Harvey Cushing/John Hay Whitney Medical Library, Yale University, New Haven, CT United States; 4 Global Health Nursing, Graduate School of Biomedical and Health Sciences, Hiroshima University, Hiroshima, Japan; 5 Yale Institute for Global Health, Yale University, New Haven, CT United States

**Keywords:** Autism spectrum disorder, Parents, Digital health intervention, Psychological health, Scoping review

## Abstract

**Background:**

Research consistently shows that parents of children with autism spectrum disorder (ASD) are at a greater risk of psychological difficulties. While various interventions exist to enhance the psychological health of these parents, the potential of digital health interventions (DHIs) in this context remains underexplored.

**Objective:**

This scoping review aims to examine the availability and effectiveness of DHIs designed to support the psychological health of parents of children with ASD.

**Methods:**

A scoping review approach was used to map the available evidence. An expert medical librarian (JB) searched six major databases—(1) CINAHL, (2) Ovid EMBASE, (3) Ovid Global Health, (4) Ovid MEDLINE, (5) Ovid PsycINFO, and (6) Web of Science—to identify studies on ASD, digital health technologies, and intervention outcomes concerning the psychological health of parents of children with ASD. Searches were conducted in June 2024. Three independent reviewers (BJ, IMSB, and XP) conducted study selection and data extraction. The methods and reporting adhered to the PRISMA Extension for Scoping Reviews (PRISMA-ScR) guidelines.

**Results:**

A total of 53 studies published between 2013 and 2024 were identified, examining the effectiveness of DHIs on the psychological health of parents of children with ASD under 18 years of age. Most studies (54.7%) originated from the United States, followed by China (13.2%). In terms of intervention content, the studies primarily focus on parental skill training and support (*n* = 27), managing children’s problem behavior and health (*n* = 15), and parental psychological health and emotional regulation (*n* = 11). Regarding intervention methods, the studies were categorized into videoconferencing telehealth, e-learning telehealth, mHealth, and asynchronous telehealth, with Zoom being the most frequently used platform (*n* = 16). Psychological health outcomes assessed in the included studies were grouped into three dimensions: negative psychological aspects, positive psychological aspects, and overall well-being, with stress being the most frequently assessed variable (*n* = 37). Significant improvements were reported in 75% of non-controlled studies and 62.1% of controlled studies, supporting the effectiveness of DHIs. All 12 studies assessing long-term effects of DHIs confirmed sustained psychological benefits.

**Conclusion:**

This review demonstrates that DHIs are a promising approach for improving the psychological health of parents of children with ASD. While the mechanisms behind their effectiveness remain unclear, DHIs offer accessible, cost-effective, and impactful support. Further research is needed to focus on parents’ psychological health and emotional regulation, explore advanced technologies, incorporate positive psychological strategies, and assess both short- and long-term outcomes in order to maximize the potential of DHIs in ASD-related care.

**Supplementary Information:**

The online version contains supplementary material available at 10.1186/s40359-025-03219-5.

## Background

A growing body of evidence suggests that parents of children with autism spectrum disorder (ASD) experience significantly higher rates of mental health issues than parents of children with other developmental disabilities [1–3]. These issues often include elevated stress levels, negative emotional states, and symptoms of depression and anxiety [3–5]. When a parent’s psychological health is compromised, it can impair their decision-making, emotional regulation, behavior control, social interactions, and stress management [6], ultimately reducing their overall problem-solving capacity [7]. Moreover, the significant psychological challenges experienced by parents can contribute to adverse psychological and developmental outcomes in their children [8, 9]. Therefore, prioritizing the psychological well-being of parents of children with ASD is important, as it directly affects their capacity to support their children and has far-reaching implications for their children’s developmental and psychological outcomes.

Traditional in-person interventions for parents of children with ASD are often constrained by factors such as time, cost, and accessibility, reducing their reach and feasibility [10–12]. In contrast, digital health interventions (DHIs) provide scalable and flexible solutions, effectively addressing these barriers and enabling broader access. DHIs, including computer-based therapies, mobile applications, and wearable devices, have the potential to improve the accessibility, efficiency, and personalization of mental health care [13]. Research on DHIs for individuals with ASD has grwon rapidly, particularly following the COVID-19 pandemic [14, 15]. This growth is largely driven by advancements in digital health technologies and the perceived advantages of these interventions, including convenience, privacy, and opportunities for independent learning [16, 17].

Recent studies indicate that most DHIs focus on delivering high-quality treatment and care for children with ASD, while comparatively less attention has been given to supporting their parents. Additionally, many of these studies have primarily concentrated on training parents to enhance their children’s social skills, communication, and learning [18, 19], with relatively few addressing the parents' own health and well-being. However, supporting parents is equally critical, given the interconnected nature of parents and children outcomes [20].

Although some studies have examined the potential benefits of DHIs in reducing parenting stress and enhancing self-efficacy [21, 22], most of these interventions have been implemented in developed countries. Furthermore, findings regarding their impact on the psychological health of parents of children with ASD remain inconsistent [16, 23–25]. Therefore, there is a pressing need to explore how DHIs can directly address the psychological well-being of parents of children with ASD, thereby filling a critical gap in the current literature.

### Objectives

We conducted a scoping review to understand whether digital health interventions can promote the psychological health of parents of children with ASD. The scoping review method was chosen for its ability to clarify key concepts, identify important characteristics, and analyze knowledge gaps in emerging fields [26]. Specifically, in this scoping review, we will (1) identify the types and strategies of DHIs developed and used to support the psychological health of parents of children with ASD; (2) evaluate the primary psychological health outcomes associated with these interventions; (3) explore existing gaps, limitations, and future opportunities for developing DHIs aimed at improving the psychological health of these parents. This study will provide a comprehensive theoretical foundation for future research on the development of DHIs to address the psychological needs of parents of children with ASD.

## Methods

### Study Design

We performed a scoping review, following the framework stages proposed by Arksey and O’Malley [27] and further refined by Levac and colleagues [28]. The methods and reporting adhered to the PRISMA Extension for Scoping Reviews (PRISMA-ScR) [29] (Multimedia Appendix 1). The protocol has been accepted for publication in JMIR Research Protocols [30].

### Search strategy

The search strategy was developed by an expert medical librarian [JB] in consultation with two researchers [BJ and ZN]. Six databases were searched: (1) CINAHL, (2) Ovid EMBASE, (3) Ovid Global Health, (4) Ovid MEDLINE, (5) Ovid PsycINFO, and (6) Web of Science. The search terms included the following keywords: (1) digital health ((digital health) OR (telemedicine) OR (mobile applications) OR (internet) OR (cell phone) OR (smartphone) OR (app) OR (apps) OR (WeChat) OR (virtual) OR (digital)); (2) autism ((autism) OR (autistic) OR (autistic disorder)); (3) population ((parent) OR (father) (mother) OR (caregiver) OR (carer) OR (family) OR (families)). The search strings were converted for each database. We also manually searched the bibliographies of relevant studies using the snowball principle to identify further eligible articles. We searched for English-language publications, excluding reviews, from the earliest retrievable records relevant to the review topic through June 25, 2024. The publications were exported into EndNote 21 (Clarivate, Philadelphia, PA), deduplicated, and then uploaded into Covidence (Veritas Health Information, Melbourne, Australia). The search queries for each database are detailed in Multimedia Appendix 2.

###  Inclusion and Exclusion Criteria

Only full-text research studies published in English in peer-reviewed journals were included. Population, Intervention, Comparator, and Outcome (PICO) principles [31] guided the formulation of the inclusion and exclusion criteria using the categories population, intervention, and outcome of interest (control type was not applicable as we included a range of study types; refer to Table 1 for details). Studies were excluded if full text was unavailable, if not published in English, if they focused exclusively on children with ASD instead of their parents, if they did not use digital health technology to deliver an intervention, or if they did not assess psychological health outcomes in parents of children with ASD. We also did not include reviews. Given the novelty of the field, no restrictions were imposed on the comparator. Studies could include no control group, a non-intervention control group, or a standard treatment control group.

**Table 1:** Study inclusion criteria following the Population, Intervention, Comparison, and Outcome principles

**Population diagnosis**
● children with ASD under the age of 18 years
● biological parents, birth parents, or foster parents of children with ASD under the age of 18 years
**Type of digital health interventions**
● computer-assisted therapy
● smartphone apps
● wearable technologies
**Outcome target**
● well-being on parental psychological health
● stress, anxiety, depression, distress, fatigue, quality of life, positive think, happiness, family empowerment, hope, resilience, etc
**Study methodology**
● qualitative, randomized controlled trials, quasi-experimental studies, cohort studies, case control studies, cross-sectional surveys, pre- and post- studies, and mixed-methods studies

### Study Selection and Screening Procedures

The study selection procedure included two review stages: (1) title and abstract review and (2) full-text review. Abstracts and full-text articles were independently reviewed by 3 reviewers (BJ, IMSB, and XP) in Covidence (Veritas Health Information, Melbourne, Australia). Discrepancies will be resolved through discussion with the senior author (ZN).

### Charting the Data

We used a narrative approach to synthesize data [32]. Data were extracted to a bespoke Microsoft Excel (Microsoft Corp) spreadsheet; one author (BJ) extracted data for all studies, and a random sample of 10% of papers was reviewed by another author (XP) to check reliability. The data extracted included author, year, full citation, country, participants (number and age), digital health intervention information (name, type, platform and tool, duration), study design, comparison, psychological outcomes for parents (variables and measurement tools), findings (significant change, significant group difference and follow-up effect). The comprehensive data extraction form is available in this publication (refer to Multimedia Appendix 3).

## Results

### Study Characteristics

**Fig. 1:**
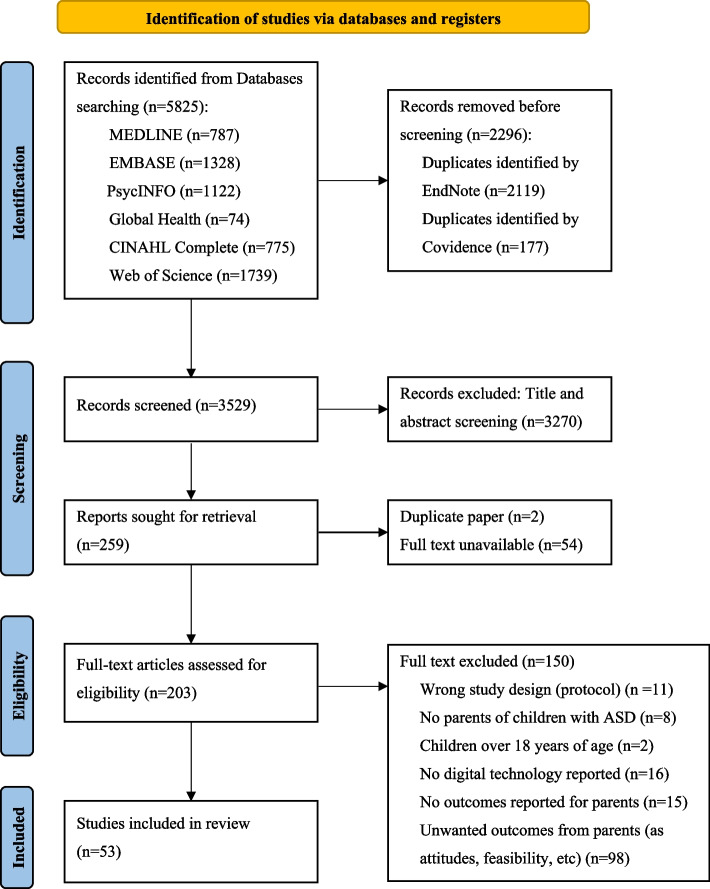
PRISMA (Preferred Reporting Items for Systematic Reviews and Meta-Analyses) flow diagram of the literature search and selection process

Our systematic search of peer-reviewed, English-language literature identified studies reporting psychological health outcomes to evaluate the effectiveness of digital health interventions supporting parents of children with ASD. Of the 259 full-text articles reviewed, 53 met the inclusion criteria, while the remaining 206 were excluded for reasons outlined in Fig. 1.

The 53 studies were published between 2013 and 2024, with a noticeable increase in publications from 2021 onwards (39 of 53 studies). The majority of studies (*n* = 29, 54.7%) originated from the United States, followed by China (*n* = 7, 13.2%) (Table 2).

**Table 2:** The main features of the included studies (*n* = 53)

Article characteristics	Value, *n*
**Publication year (*n* = 53)**	
2013	1
2014	1
2015	2
2016	1
2017	2
2018	4
2019	1
2020	2
2021	7
2022	13
2023	10
2024	9
**Country of origin (*n* = 53)**	
United States	29
China	7
Italy	3
Canada	2
Australia	2
Iran	2
Japan	2
South Korea	2
India	1
Netherlands	1
Saudi Arabia	1
Indonesia	1

### Characteristics of Participants

The participants included parents of children with ASD or parent–child dyads involving a child with ASD. The sample size across the studies ranged from one to 263 participants. Of the 53 studies included, 39 involved both father and mother, 13 focused exclusively on mothers, and one focused solely on fathers. Collectively, findings were reported for 2649 parents of children with ASD. Regarding parental age, 20 studies included parents under 40 years of age, 8 studies reported on parents aged 40 years or older, and 25 studies did not specify the parents’ ages (Table 3). Among the studies, 32 included parents of children under 6 years old, 14 included children aged 6 years and older, and 7 studies provided only the age range of the children, without specific average age (Table 3). No studies involved parents of children over 18 years.

**Table 3:** Study populations (n = 53)

Article characteristics	Value, *n*
**Participant Type (** ***n*** ** = 53)**	
Parent–child dyad	29
Parent only	24
**Parents of Children with ASD**	
Sample size (range 1–263)	
< 50-	32
50–100	14
> 100	7
Gender (*n* = 53)	
Mother and father	39 (specified-28, unspecified-11)
Mother only	13
Father only	1
Age (*n* = 53)	
< 40 years	20
≥ 40 years	8
NR^a^	25
Total parents	2,649
**Children with ASD**	
Age (*n* = 53)	
≤ 6 years	32
> 6 years	14
NR^a^	7

^a^NR: Not reported

### Intervention Characteristics

From the perspective of intervention content, the interventions primarily focus on parental skill training and support (*n* = 27), parental psychological health and emotional regulation (*n* = 11), as well as managing children’s problem behaviors and health (*n* = 15). From the perspective of intervention methods, the interventions were heterogeneous and categorized into four main types: videoconferencing telehealth (*n* = 37), e-learning telehealth (*n* = 10), mHealth (*n* = 7), and asynchronous telehealth (*n* = 4), with some interventions combining more than one of these approaches. The most commonly used digital intervention platform or tool was video conferencing software, with Zoom being the most frequently used one (*n* = 16). The intervention duration ranged from 3 to 50 weeks, with the average intervention period for the 51 studies being 10.87 weeks, excluding two studies where the duration was reported as a range.

The included studies comprised 23 randomized controlled trials (RCTs), four non-randomized controlled trials (non-RCTs), 22 cohort studies with pre- and post-intervention (PPI) designs, one pretest–posttest control group design, two case reports, and one cross-sectional study (Table 4). Among the 27 studies with controlled designs, four included three or more comparison groups, while the remaining studies utilized two-group comparisons. Specifically, 21 studies evaluated the effectiveness of digital health interventions against control groups, including waitlist control groups (WLC), treatment-as-usual (TAU) groups, active control groups (ACG), or no-treatment control groups (NTCG). Additionally, eight studies compared the effectiveness of digital health interventions with in-person groups (IPG), while three studies compared digital health interventions to printed material control groups. Regarding intervention types, six studies utilized two intervention types, 46 studies each employed a single intervention type, and one study did not provide specific details.

**Table 4:** Summary of studies on the effectiveness of digital health interventions for psychological health in parents of children with ASD (*n* = 53)

Reports	Digital health intervention information				Study design	Comparison	Psychological outcomes for parents	Findings		
	Name	Type	platform and tool	Duration (weeks)				Significant change	Significant group difference	Follow-up effect (weeks)
Andrews et al. [33]	Acceptance and commitment training (ACT) + behavior parent training (BPT)	Telehealth-videoconference + Asynchronous Telehealth	Yondo (a HIPAA compliant real-time video-conferencing platform) + email	11	PPI^a^ design	None	Perceived stress Experiential avoidance	Yes	None	Yes (1 and 4)
Batton et al. [25]	The Online and Applied System of Intervention Skills (OASIS)	Telehealth-elearning + videoconference	NR^b^	16	PPI design	None	Parental stres Quality of life Self-efficacy	Yes	None	None
Bekhet et al. [24]	Positive thinking training (PTT)	Telehealth-elearning	A course named"intervention for caregivers"on the website: https://cps.ce.mu.edu	6	RCT^c^	NTCG^d^	Positive thinking	Yes	NR	None
Clifford et al. [34]	Online Parent Support Group (PSG)	Telehealth-videoconference + Asynchronous Telehealth	Real-time online chat sessions + email	8	Non-RCT	IPG^e^	Stress Anxiety Depression Positive perception	No	No	None
Curl et al. [35]	Virtual Mindful Self-Compassion (MSC) workshop	Telehealth-videoconference	A video conferencing software	4	PPI design	None	Parenting stress Self-efficacy Mindfulness Self-Compassion	Yes	None	None
Fenning et al. [36]	Mindfulness-Based Stress Reduction (MBSR) virtual or psychoeducational support (PE) virtual intervention	Telehealth-videoconference	Zoom	8	RCT	IPG (MBSR or PE)	Parental stress Stress specific to the autistic child Daily parenting hassles	No	NR	Yes (24 and 48)
Ferrante et al. [37]	Virtual WHO Caregiver Skills Training (CST)	Telehealth-videoconference	Zoom and Webex + smartphone, laptop or tablet	9	RCT	IPG and TAUf	Parental stress Self-efficacy Competence	Yes: competence only	Yes: competence only	None
Garnett et al. [38]	Hanen More Than Words (HMTW) intervention	Telehealth-videoconference	Zoom + desktop computers, laptops, or tablets	11	PPI design	None	Parenting stress	No	None	None
Gentile et al. [39]	ATHENA telehealth program	Telehealth-videoconference	ATHENA App, tablet and bluetooth headset	24	PPI design	None	Parenting stress Empowerment	Yes	None	None
Gould et al. [40]	Research Units in Behavioral Intervention via teleheath (RUBI-T)	Telehealth-videoconference	Zoom and webcam-enabled laptop computers	25.5	PPI design	None	Parenting stress	Yes	None	Yes (12)
Hajiabolhasani-Nargani et al. [41]	mobile parenting skills education on anxiety	Mobile health (mHealth)	Mobile phone and interactive text messaging	8	RCT	PMCG^g^	Anxiety	Yes	Yes	None
Hemdi et al. [42]	Psychoeducation Intervention	Mobile health (mHealth)	WhatsApp, visual and audio media	5	RCT	TAU	Parental stress Anxiety and depression Happiness	Yes: parental stress, depression and happiness only	Yes: parental stress, depression and happiness only	Yes (8)
Ibañez et al. [43]	Web-based enhancing interactions tutorial	Telehealth-elearning	Web-based tutorial + videos	4	RCT	WLCh	Parenting stress Efficacy	No	No	Yes (4)
Ingersoll et al. [44]	Self-directed ImPACT Online (SD) or therapist-assisted ImPACT Online (TA)	Telehealth-elearning or videoconference	Zoom	24	RCT	PMCG	Parenting stress Competence Positive impact	Yes: positive impact only	Yes: positive impact only	Yes (12)
Inoue et al. [45]	Internet based parent education (PE) program	Telehealth-elearning	General empowerment tool for abilities (GETA) + PPT, lecture, e-mail	4	PPI design	None	Mental health status	Yes	None	None
Inoue et al. [46]	Online Parent Training (PT)	Telehealth-elearning	PC and smartphone + email	9	Case report	None	Stress Deprssion	Yes	None	None
Iovino et al. [47]	Facebook group self-care intervention	Mobile health (mHealth)	Facebook	8	PPI design	None	Stress	Yes	None	None
Ip et al. [48]	Parent-based sleep-focused intervention	Telehealth-videoconference	Zoom	8	RCT	TAU	Depression, anxiety and stress Parental stress Competence	NR	Yes: stress only	None
Jamali et al. [49]	Occupationa performance coaching (OPC)	Telehealth-videoconference	WhatsApp software video calling feature	8	RCT	WLC	Quality of life Self-efficacy	Yes	Yes: self-efficacy only	Yes (6): self-efficacy only
John et al. [50]	Engaged Eaters Program–Telehealth	Telehealth-videoconference	Zoom	24	PPI design	None	Stress Competence	No	None	None
Johnson et al. [51]	an iPad application (app) social script intervention	Mobile health (mHealth)	iPad app	3	RCT	TAU	Anxiety	NR	Yes	None
Johnsonet al [52]	Sleep parent training (SPT)	Telehealth-videoconference	Zoom + email	10	RCT	ACG^i^ (SPE)	Stress Competence	NR	Yes: competence only	None
Kangavary et al. [53]	Coping Options for Parent Empowerment (COPE)	Telehealth-videoconference	NR	4	Case report	None	Anxiety Depressive Competentce Hope	Yes	None	None
Kenworthy et al. [54]	Online executive function training	Telehealth-videoconference	3 C Institute’s proprietary Dynamic e-Learning Platform (DeLP; 3cisd.com/e-learning-for-behavior-change)	10	RCT	IPG	Strain Empowerment	Yes	Yes	None
Kuhlthau et al. [55]	Stress management and resiliency training-relaxation response resiliency program (SMART-3R)	Telehealth-videoconference	HIPAA-compliant online group videoconferencing program	8	RCT	WLC	Distress Resiliency Worry Parental stress Depression and anxiety Distress Positive affect Mindfulness	Yes: distress, resiliency, parental stress, depression/anxiety, positive affect, and mindfulness only	Yes: resiliency, parental stress, depression/anxiety, and mindfulness only	Yes (12): distress, resiliency, parental stress, depression/anxiety, positive affect, and mindfulness only
Kuravackel et al. [56]	COMPASS for Hope (C-HOPE)	Telehealth-videoconference	NR	8	Pretest–Posttest Control Group Design	IPG and WLC	Stress Competence	Yes	No	None
Lau et al. [57]	WHO-caregiver skills training (CST) Program	Telehealth-elearning or videoconference	Digital versions of CST participant booklets and pre-recorded videos, and zoom	8	RCT	IPG and WLC	Quality of life	Yes	Yes	None
Lee et al. [58]	Challenging Behavior Online Modules (CBOM)	Telehealth-elearning	Website and videos with audio narrative	3	RCT	WLC	Stress	Yes	Yes	None
Lestari et al. [59]	Online counseling	NR (Synchronous Telehealth)	NR	4	PPI design	None	Anxiety	Yes	None	None
Li et al. [60]	Online-delivered Project ImPACT	Telehealth-videoconference	Tencent Meeting Application (Tencent Corp, Shanghai) and WeChat	8	Non-RCT	WLC	Stress Competence	Yes	Yes	None
Little et al. [61]	Occupation-Based Coaching	Telehealth-videoconference	Zoom	12	PPI design	None	Competence	Yes	None	None
Liu et al. [62]	WeChat-based parenting training	Mobile health (mHealth)	WeChat	12	Non-RCT	TAU	Stress Anxiety Hope	Yes	Yes	Yes (8)
Malow et al. [63]	A REDCap-based model for online intervention	Telehealth-elearning	Online digital platform (www.autismspeaks.org/tool-kit/atnairp-strategies-improve-sleep-children-autism), multimedia materials, videos, tablet, computers or smartphones	12	RCT	PMCG	Competence	No	No	None
Marino et al. [64]	Tele-assisted behavioral intervention	Telehealth-videoconference	A web platform, providing video conference tools (https://gsuite.google.com)	12	RCT	IPG	Stress	Yes	NR	None
Martin et al. [65]	Behavioral parent training (BPT) program	Telehealth-videoconference	Zoom	20	PPI design	None	Stres Competence	Yes	None	None
May et al. [66]	SMS (Text2dads) Intervention	Mobile health (mHealth)	Text messages, short films, online service shorten links (BITLY)	16	Cross sectional study	None	Parental stress Competence Self-efficacy	Yes: parenting stress and self-efficacy only	None	None
Montiel-Nava et al. [67]	WHO-caregiver skills training (CST) program	Telehealth-videoconference	Extension for community healthcare outcomes (ECHO) model, zoom, desktop computer, laptop, or tablet with a video camera + videos	12	PPI design	None	Stress Distress Psychological distress Competence	No	None	None
Neely et al. [68]	Caregiver-implemented behavioral intervention	Telehealth-videoconference	VSee software (VSee, 2021), eliademy and OneDrive	5	PPI design	None	Competence	Yes	None	None
O'Brien et al. [69]	Functional communication training (FCT)	Telehealth-videoconference	Computers equipped with Vidyo or Skype and Debut video capture software, windows based PC and video monitor	8–24	PPI design	None	Stress	Yes	None	None
Pandya et al. [70]	WhatsApp-based spiritual posts intervention	Mobile health (mHealth)	Whatsapp, google group	50	RCT	WLC	Stress Self-efficacy Confidence Resilience	Yes	Yes	None
Pennefather et al. [71]	Online autism parent training (APT)	Telehealth-elearning + videoconference	Google + hangouts, and google + community page	3	PPI design	None	Stress	No	None	None
Poslawsky et al. [72]	Video-feedback intervention to promote positive parenting adapted to autism (VIPP-AUTI)	Asynchronous Telehealth	Videos	12	RCT	TAU	Self -efficacy	Yes	Yes	Yes (12)
Qi et al. [73]	Online Hanen More Than Words (HMTW) program	Telehealth-videoconference	Zoom	12	RCT	TAU	Parenting stress Self-efficacy	Yes: self-efficacy only	Yes: self-efficacy only	None
Roberts et al. [74]	Online Parent Education	Telehealth-videoconference	Online learning platforms (Moodle), and podcasts and blogs, PowerPoint script, and email	4	Non-RCT	IPG	Quality of life Fatigue	Yes	Yes	None
Rodgers et al. [75]	Telemedicine parent-delivered social skills intervention (PDSSI)	Telehealth-videoconference	HIPAA-compliant video conference platform	4	PPI design	None	Stress Empowerment	Yes	None	Yes (16)
Ros-DeMarize et al. [76]	Tele-Parent–Child Interaction Therapy (PCIT)	Telehealth-videoconference	Doxy.me secure web-based platform	10	PPI design	None	Stress	Yes	None	Yes (12)
Vismara et al. [77]	Early start denver model (ESDM) parent coaching	Telehealth-videoconference	multimodal learning modalities (e.g., text, video, and audio), HIPAA-compliant video-conferencing software, and laptop, tablet, or smartphone	24	PPI design	None	Competence	Yes	None	None
Wainer et al. [78]	Stepped-care model of Online RIT (reciprocal imitation training)	Telehealth-videoconference	Vidyo	10	RCT	TAU	Family quality of life Self-efficacy	Yes: self-efficacy only	Yes: self-efficacy only	None
Wallisch et al. [79]	Ecological momentary assessment (EMA)	Telehealth-videoconference	HIPAA-compliant videoconferencing software, and zoom	8	PPI design	None	Parental stress Perceived stress Self-efficacy	Yes: self-efficacy and parental stress only	None	None
Whitney et al. [80]	Emotional Disclosure Through Journal Writing: Telehealth Intervention	Asynchronous Telehealth	Online journal writing program	8	RCT	WLC	Stress	No	No	None
Yeom et al. [81]	Responsive teaching (RT) curriculum	Telehealth-videoconference	Zoom	8–16	PPI design	None	Stress	Yes	None	None
Zhao et al. [82]	Web-based parent–child physical activity program	Telehealth-videoconference	Youth Sport, WeChat, Zoom or Tencent Live	10	RCT	TAU	Stress Depression, anxiety and stress Quality of life	Yes	Yes	None
Zhao et al. [83]	web-based 24-h movement behavior lifestyle education program	Telehealth-videoconference	Web-based program featuring teaching videos and live workshops, and WeChat	8	RCT	WLC	Depression, anxiety and stress Life satisfaction	Yes	Yes	None

^a^PPI: Pre- and Post-Intervention

^b^NR: Not reported

^c^RCT: Randomized controlled trial

^d^NTCG: No-treatment control group

^e^IPG: In-person group

^f^TAU: Treatment as usual

^g^PMCG: Printed material control group

^h^WLC: Waitlist control group

iACG: Active control group

### Measures

The 53 included studies reported at least one psychological health variable, and 20 distinct variables were used to assess parents’ psychological health (Table 5). The psychological health variables can be categorized into three main dimensions: (1) negative psychological dimensions, encompassing stress, anxiety, depression, fatigue, worry, and related constructs; (2) positive psychological dimensions, including competence, self-efficacy, positive affect and cognition, empowerment, hope, mindfulness, happiness, and confidence; and (3) comprehensive psychological states, including quality of life. The most studied variable was stress, which was examined in 37 of the 53 studies. Specific dimensions of stress varied across studies, including parenting stress, parental stress, perceived stress, general stress, and stress specifically associated with parenting children with ASD. In addition to stress, competence and self-efficacy were frequently investigated. Notably, some studies used these terms interchangeably, as certain measurement tools, such as the Parenting Sense of Competence Scale, include self-efficacy as a subscale.

**Table 5:** Outcomes and measurement tools for the psychological health of parents of children with ASD (*n* = 53)

Dimensions	Outcomes	Value, *n*	Measurement tools
Negative psychological dimension	Stress	37	Parenting Stress Index-long form & short form (PSI) [84] Parental Stress Scale (PSS) [85–87] Perceived Stress Scale (PSS-10) [88] Autism Parent Stress Index (APSI) [6] CAPES Parental Self-Efficacy Scale (CAPES-PSES) [89] Family Stress and Coping Interview (FSCI) [90] Negative Impact scale of the Family Impact Questionnaire (FIQ) [91] The Intensity subscale of the Parenting Daily Hassles (PDH) [92] Depression Anxiety and Stress Scale (DASS-21) [93]
	Anxiety	11	Depression Anxiety and Stress Scale (DASS-21) [93] Generalized Anxiety Disorders-7 (GAD-7) [94] Spielberger Anxiety Inventory (SAI) [95] Self-rating Anxiety Scale (SAS) [96] State Trait Anxiety Inventory (STAI) [97] Hospital Anxiety and Depression Scale (HADS) [98] Patient Health Questionnaire-4 (PHQ-4) [99]
	Depression	8	Depression Anxiety and Stress Scale (DASS-21) [93] Patient Health Questionnaire-4/9 (PHQ-4/9) [100, 101] Beck Depression Index II (BDI-II) [102] State-Trait Depression Scales (STDS) [103] Hospital Anxiety and Depression Scale (HADS) [98]
	Distress	2	Brief Family Distress Scale (BFDS) [104] The Kessler Screening Scale for Psychological Distress (K6) [105] Visual Analogue Scale (VAS) [106]
	Fatigue	1	Fatigue Assessment Scale (FAS) [107]
	Worry	1	Penn State Worry Questionnaire (PSWQ) [108]
	Strain	1	The Caregiver Strain Questionnaire-Short Form 7 (CGSQ-SF7) [109]
	Experiential avoidance	1	The Parental Acceptance and Action Questionnaire (PAAQ) [110]
Positive psychological dimension	Competence	16	Parenting Sense of Competence Scale (PSOC) [111, 112] Co-parenting Competence Scale (CCS) [113] Knowledge and Skills Questionnaire (KSQ) (WHO CST Team, unpublished)
	Self-efficacy	10	Parenting Sense of Competence Scale (PSOC) [111, 114] Early Intervention Parenting Self-Efficacy Scale (EIPSES) [115] Autism Parenting Questionnaire (APQ) [116] Caregiver Self-efficacy Questionnaire (CSQ) (WHO CST Team, unpublished) Parental efficacy questionnaire (PEQ) [117] Parental efficacy scale (PES) [118] Child adjustment and parent efficacy scale-developmental disability (CAPES-DD) [99]
	Positive affect and cognition	4	Positive Impact subscale of the Family Impact Questionnaire (FIQ) [91] Positive and Negative Affect Schedule‑Positive Subscale (PANAS‑P) [119] Positive Thinking Skills Scale (PTSS) [120] Kansas Inventory of Parental Perceptions (KIPP) [121]
	Empowerment	2	Family Empowerment Scale (FES) [122]
	Hope	2	State Hope Scale (SHS) [123]
	Mindfulness	2	Freiburg Mindfulness Inventory (FMI) [124] Cognitive and Affective Mindfulness Scale-Revised (CAMS-R) [125]
	Resilience	2	Current Experience Scale (CES) [126] Parenting Resilience Elements Questionnaire (FREQ) [127]
	Happiness	1	Arabic Scale of Happiness [128]
	Confidence	1	Maternal Confidence Questionnaire (MCQ) [129]
	Self-compassion	1	Self-compassion Scale (SCS) [130]
	Life satisfaction	1	The Satisfaction With Life Scale (SWLS) [131]
Comprehensive psychological state	Quality of life	6	Short-Form Health Survey (SF-36) [132] WHO Quality of Life Assessment-26 (WHOQOL-26) [133] Family quality of life (FQOL) Scale [134] General Health Questionnaire (GHQ-12/30) [135, 136] PedsQL Inventory (Family Impact Module 2.0) [137]

### Results Summary

Most included studies showed statistically significant outcomes, demonstrating the effectiveness of DHIs in reducing negative psychological factors, improving positive psychological factors, and enhancing overall psychological well-being (Table 4). Among the 24 studies without a control group, 18 (75.0%) reported significant improvements in psychological outcomes following DHIs, two (8.3%) demonstrated partial effectiveness for specific variables, and four (16.7%) found no significant changes. Of the 29 studies employing a control group design, 18 studies (62.1%) demonstrated significant improvements from pre- to post-intervention within the treatment group as well as significant differences between intervention and control groups, reaffirming the overall effectiveness of the interventions. Six studies (20.7%) showed partial positive effects: 3 (10.3%) reported significant changes only from pre- to post-intervention, while 3 (10.3%) found significant differences exclusively between groups. Five studies (17.2%) found no significant differences in any comparisons. Additionally, all 12 studies assessing the long-term effects of DHIs on the psychological health of parents of children with ASD confirmed their effectiveness.

## Discussion

### Principal Findings

This scoping review presents a comprehensive overview of the existing evidence on DHIs aimed at improving the psychological health of parents of children with ASD, with a specific focus on their availability and effectiveness. Publication trends indicate a rapid growth in this field over the past four years, reflecting increasing interest in this area of research [138]. A higher volume of studies originates from the United States and China, likely due to their robust economic resources and advanced technological infrastructure [139, 140]. Our review analyzed 53 research papers, involving a total of 2,649 parents of children with ASD. The research findings provide preliminary evidence supporting the availability and effectiveness of DHIs for this population.

In examining the availability of DHIs, we found that only 11 studies focused specifically on parental mental health and emotional regulation. This limited focus may be due to the assumption that improving children's behavior and parenting skills indirectly benefits parents [141]. However, parental mental health often requires direct support [142]. This gap highlights the need for more targeted interventions that directly address parental mental health. Future research should prioritize developing and implementing interventions to ensure that parents not only gain the necessary skills and support to care for their children but also develop the emotional resilience required to manage the stress associated with raising a child with ASD. Additionally, our analysis revealed a wide variety of DHI formats, including videoconferencing telehealth, e-learning telehealth, mHealth, and asynchronous telehealth. Among these formats, videoconferencing telehealth was most widely used, with Zoom being the most commonly employed platform. These findings confirm that DHIs are available for improving the psychological health of parents of children with ASD. However, they also reveal certain limitations in the current application of DHIs. Firs, videoconferencing telehealth primarily serves as an enhancement to traditional intervention methods by improving delivery formats and addressing accessibility challenges related to time and location [143]. To further develop intervention content and improve the overall intervention environment, the integration of more advanced DHI technologies, such as virtual reality (VR), augmented reality (AR), and artificial intelligence (AI), should be encouraged for use in this field [144, 145]. Second, we found that the psychological health variables targeted by DHIs for parents of children with ASD can be categorized into three main dimensions: negative psychological dimensions, positive psychological dimensions, and overall psychological well-being. Among them, the most frequently studied variable was the negative psychological dimension, particularly stress, which was addressed in 37 out of the 53 studies. We recommend focusing on positive outcomes should assess not only reduced distress but also enhanced psychological strengths and well-being [146]. More research should further explore how to the incorporate positive psychological elements into DHIs [147], such as enhancing quality of life, fostering resilience, and promoting self-efficacy and hope [25, 53, 70].

In evaluating the effectiveness of DHIs, the accumulated evidence indicates their positive impact on improving the psychological health of parents of children with ASD. Among the 24 studies without a control group, 18 reported significant improvements from pre- to post-intervention. Similarly, of the 29 studies with a control group design, 18 demonstrated significant improvements both within the intervention group and between the intervention and control groups. Furthermore, our review confirms the long-term benefits of DHIs in enhancing the psychological health of this population [36, 40, 42–44, 62, 72, 75, 76]. However, the specific reasons behind the lack of significant intervention effects in nine studies remain unclear. Possible contributing factors may include the selection of study participants, inconsistencies in the intervention protocols, insufficient intervention duration, and the choice of outcome variables or measurement tools [34, 36, 38, 43, 50, 63, 67, 71, 80]. Interestingly, although two studies did not report statistically significant differences either from pre- to post-intervention or between the intervention and control groups, they did reveal the long-term effects of DHIs [36, 43]. The mechanisms underlying the effectiveness of DHIs in improving the psychological health of parents of children with ASD are not yet well understood, posing a challenge to draw generalizable conclusions.

Despite these uncertainties, we remain optimistic about the potential of DHIs in supporting the psychological health of parents of children with ASD. We believe that substantial and growing evidence in the future will emerge to support the effectiveness of DHIs for ASD. As digital health technologies and applications become increasingly popular and AI advances [144, 145], the integration of evidence-based psychological treatments with digital content makes DHIs promising, effective, cost-effective, and accessible care for individuals with ASD and their caregivers.

### Future Research Agenda on DHIs for ASD

We advocate for further research to advance this field and propose several considerations for researchers to address in future studies and clinical practice. First, beyond the commonly used telehealth video conferencing, the design and implementation of DHIs for parents of children with ASD should carefully evaluate the potential benefits and opportunities offered by various advanced DHI technologies, such as virtual reality (VR), augmented reality (AR), artificial intelligence (AI), and wearable devices. These advanced technologies can not only enhance digital health, streamline health care, and personalized health services, but also personalize benefits and intelligence solutions [148]. Additionally, they can improve user motivation, maximize learning efficiency, and offer security solutions tailored to the specific needs of parents [149]. Second, DHI research should consider the effects of interventions from both negative and positive psychological health perspectives. Studies show interventions designed with a positive psychology lens can have a substantial positive impact, leading to improved outcomes in various areas of well-being [150]. Third, given the life-long nature of parental care for children with ASD and the lack of reliable data on the long-term effects and sustained impact of DHIs, more future research should explore both short- and long-term treatment outcomes. Finally, due to the limited evidence on DHIs for the psychological health of parents of children with ASD, clinicians should be cautious in recommending them. Future research should explore not only the impact of delivery modes—such as remote versus in-person interventions—but also the underlying psychological and neurobiological mechanisms that contribute to their effectiveness.

### Limitations

This review adopted a broad scope to capture a wide range of studies, including varied subgroup populations, DHI approaches, and psychological health variables. Given the early stage of research in this field, this approach was appropriate for providing a preliminary overview of the available literature, but future systematic investigations should adopt a more focused approach to validate our conclusions and strengthen the evidence base, particularly concerning subgroups, types of DHIs, and psychological health variables relevant to parents of children with ASD. Additionally, future research would benefit from incorporating the perspectives of stakeholders and parents. The inclusion of both experimental and observational studies in this review may have introduced methodological limitations, particularly regarding causal inference. Furthermore, due to the lack of quality assessment standards for digital mental health interventions [151], we did not exclude studies based on the potentially suboptimal quality of the digital applications used. Another limitation is the variability in diagnostic methods across the included studies, as many studies did not explicitly specify how autism diagnosis was confirmed, which may impact the consistency and reliability of the findings. Moreover, feasibility studies suggest that factors such as digital literacy, internet access, and privacy concerns may affect the implementation and effectiveness of DHIs, which should be taken into account when interpreting the generalizability of the results.

## Conclusions

This scoping review highlights the growing body of evidence supporting the effectiveness and availability of DHIs aimed at improving the psychological health of parents of children with ASD. Despite some uncertainties regarding the mechanisms of effectiveness, DHIs show promise in providing accessible, cost-effective, and impactful support for this population. The review underscores the need for further research in several key areas: direct focus on parents’ psychological health and emotional regulation, exploration of advanced technologies, integration of positive psychological aspects into interventions, examination of both short- and long-term outcomes, and investigation of the mechanisms underlying DHI effectiveness. With continued advancements in digital technologies, including AI, machine learning, and AR, DHIs hold significant potential to enhance the well-being of parents and address critical gaps in ASD-related care.

## Supplementary Information

Supplementary Material 1. Multimedia Appendix 1. The PRISMA-ScRchecklist.

Supplementary Material 2. Multimedia Appendix 2. Search strategies.

Supplementary Material 3. Multimedia Appendix 3. Data extraction form.

## Data Availability

No datasets were generated or analysed during the current study.

## Abbreviations

ACG: Active control group
ASD: Autism spectrum disorders
DHI: Digital health intervention
IPG: In-person group
mHealth: Mobile health
NTCG: No-treatment control group
NR: Not reported
PICO: Population, Intervention, Comparator, Outcomes
PMCG: Printed material control group
PPI: Pre- and Post-Intervention
PRISMA: Preferred Reporting Items for Systematic Reviews and Meta-Analyses
RCT: Randomized controlled trial
TAU: Treatment as usual
WHO: World Health Organization
WLC: Waitlist control group
