# Supplementary material for: Digital health interventions targeting psychological health in parents of children with autism spectrum disorder: a scoping review
Source: BMC Psychol. 2025 Oct 10;13:1128. doi: 10.1186/s40359-025-03219-5 (PMC12512936; doi:10.1186/s40359-025-03219-5)
Supplement: Supplementary file 2 — Supplementary Material 2. Multimedia Appendix 2. Search strategies. [file 40359_2025_3219_MOESM2_ESM.docx]

**Multimedia Appendix 2: Search strategies**

OVID MEDLINE (R) ALL

| 1 | exp Telemedicine/ |
| --- | --- |
| 2 | exp Digital Health/ or exp Internet/ or exp Mobile Applications/ or exp Telemedicine/ or exp Cell Phone/ or exp Smartphone/ |
| 3 | (Smartphone* or App or apps or WeChat or Text* or Virtual* or telehealth or digital or cellphone* or cell phone*).ti,ab,kw. |
| 4 | 2 or 3 or 4 |
| 5 | (autism or autistic).mp. or exp Autistic Disorder/ |
| 6 | 5 and 6 |
| 7 | exp Parents/ |
| 8 | exp Fathers/ |
| 9 | exp Mothers/ |
| 10 | exp Caregivers/ |
| 11 | exp Family/ |
| 12 | (parent* or father* or mother* or caregiver* or carer* or family or families).ti,ab,kw. |
| 13 | 8 or 9 or 10 or 11 or 12 or 13 |
| 14 | 7 and 14 |
| 15 | review.m_titl. |
| 16 | 15 not 16 |
|  |  |

EMBASE

| 1 | exp telemedicine/ |
| --- | --- |
| 2 | exp digital technology/ |
| 3 | exp Internet/ |
| 4 | exp mobile application/ |
| 5 | exp telemedicine/ |
| 6 | exp mobile phone/ |
| 7 | exp smartphone/ |
| 8 | (Smartphone* or App or apps or WeChat or Text* or Virtual* or telehealth or digital or cellphone* or cell phone*).ti,ab,kw. |
| 9 | 1 or 2 or 3 or 4 or 5 or 6 or 7 or 8 |
| 10 | exp autism/ or autism.mp. or autistic.mp. [mp=title, abstract, heading word, drug trade name, original title, device manufacturer, drug manufacturer, device trade name, keyword heading word, floating subheading word, candidate term word] |
| 11 | exp parent/ |
| 12 | exp father/ |
| 13 | exp mother/ |
| 14 | exp caregiver/ |
| 15 | exp family/ |
| 16 | (parent* or father* or mother* or caregiver* or carer* or family or families).ti,ab,kw. |
| 17 | 11 or 12 or 13 or 14 or 15 or 16 |
| 18 | 9 and 10 and 17 |
| 19 | review.m_titl. |
| 20 | 18 not 19 |
|  |  |

OVID APAPsycINFO

| 1 | exp Telemedicine/ |
| --- | --- |
| 2 | exp Digital Technology/ |
| 3 | exp Internet/ |
| 4 | exp Mobile Applications/ |
| 5 | exp Telemedicine/ |
| 6 | exp Mobile Phones/ |
| 7 | exp Smartphones/ |
| 8 | (Smartphone* or App or apps or WeChat or Text* or Virtual* or telehealth or digital or cellphone* or cell phone*).ti,ab. |
| 9 | 1 or 2 or 3 or 4 or 5 or 6 or 7 or 8 |
| 10 | (autism or autistic).mp. or exp Autism Spectrum Disorders/ |
| 11 | exp Parents/ |
| 12 | exp Fathers/ |
| 13 | exp Mothers/ |
| 14 | exp Caregivers/ |
| 15 | exp Family/ |
| 16 | (parent* or father* or mother* or caregiver* or carer* or family or families).ti,ab. |
| 17 | 11 or 12 or 13 or 14 or 15 or 16 |
| 18 | 9 and 10 and 17 |
| 19 | review.m_titl. |
| 20 | 18 not 19 |
|  |  |

OVID Global Health

| 1 | telemedicine/ |
| --- | --- |
| 2 | digital technology/ |
| 3 | internet/ |
| 4 | mobile applications/ |
| 5 | telemedicine/ |
| 6 | mobile telephones/ |
| 7 | (Smartphone* or App or apps or WeChat or Text* or Virtual* or telehealth or digital or cellphone* or cell phone*).ti,ab. |
| 8 | 1 or 2 or 3 or 4 or 5 or 6 or 7 |
| 9 | (autism or autistic).mp. |
| 10 | parents/ |
| 11 | fathers/ |
| 12 | mothers/ |
| 13 | careproviders.sh. |
| 14 | families/ |
| 15 | (parent* or father* or mother* or caregiver* or carer* or family or families).ti,ab. |
| 16 | 10 or 11 or 12 or 13 or 14 or 15 |
| 17 | 8 and 9 and 16 |
|  |  |

CINAHL Complete

| S1 | (MH "Telemedicine+") OR (MH "Telehealth+") |
| --- | --- |
| S2 | (MH "Digital Technology+") OR (MH "Digital Health+") |
| S3 | (MH "Internet+") |
| S4 | (MH "Mobile Applications") |
| S5 | (MH "Cellular Phone+") |
| S6 | (MH "Smartphone") OR (MH "Text Messaging+") |
| S7 | Smartphone* or App or apps or WeChat or Text* or Virtual* or telehealth or digital or cellphone* or cell phone* |
| S8 | S1 OR S2 OR S3 OR S4 OR S5 OR S6 OR S7 |
| S9 | (MH "Autistic Disorder") OR ("autism" OR "autistic") |
| S10 | (MH "Parents+") |
| S11 | (MH "Fathers+") |
| S12 | (MH "Mothers+") |
| S13 | (MH "Caregivers") |
| S14 | (MH "Family+") |
| S15 | parent* or father* or mother* or caregiver* or carer* or family or families |
| S16 | S10 OR S11 OR S12 OR S13 OR S14 OR S15 |
| S17 | S8 AND S9 AND S16 |
| S18 | TI "review" |
| S19 | S17 NOT S18 |
|  |  |
|  |  |
|  |  |
|  |  |
|  |  |
|  |  |
|  |  |
|  |  |
|  |  |
|  |  |

Web of Science

|  |  |
| --- | --- |
| 4 | **#1 AND #2 AND #3** |
| 3 | **((((((ALL=(parent*)) OR ALL=(father*)) OR ALL=(mother*)) OR ALL=(caregiver*)) OR ALL=(carer*)) OR ALL=(family)) OR ALL=(families)** |
| 2 | **(TS=(autism)) OR TS=(autistic)** |
| 1 | **(TS=(telemedicine) OR TS=(digital health) OR TS=(internet) OR TS=(mobile application*) OR TS=(smartphone*) OR TS=(cell phone*) OR TS=(apps) OR TS=(app) OR TS=(mobile phone*) OR TS=(wechat) OR TS=(text*) OR TS=(virtual) OR TS=(telehealth) OR TS=(digital))** |
